# To vaccinate or not to vaccinate patients undergoing biologic treatment in dengue-endemic countries?

**DOI:** 10.1016/j.jdin.2024.07.009

**Published:** 2024-08-10

**Authors:** María Julia Cura, Luis Daniel Mazzuoccolo

**Affiliations:** Department of Dermatology, Hospital Italiano de Buenos Aires, Buenos Aires, Argentina

**Keywords:** biologics, dengue vaccines

*To the Editor:* During the last summer, several patients undergoing biologic treatments who contracted dengue asked: do I need to receive the new dengue vaccine? We were at a loss for words.

Dengue is a viral infection in tropical and subtropical areas, primarily transmitted by *Aedes* mosquitoes. Since 2009, dengue has been classified as symptomatic dengue without significant complications and severe dengue if individuals experience complications.^1^

In 2023, approximately 5 million new dengue cases and more than 5000 deaths were reported globally, including in Africa, the Americas, Southeast Asia, Western Pacific, and Eastern Mediterranean Regions (Fig 1).

**Fig 1:**
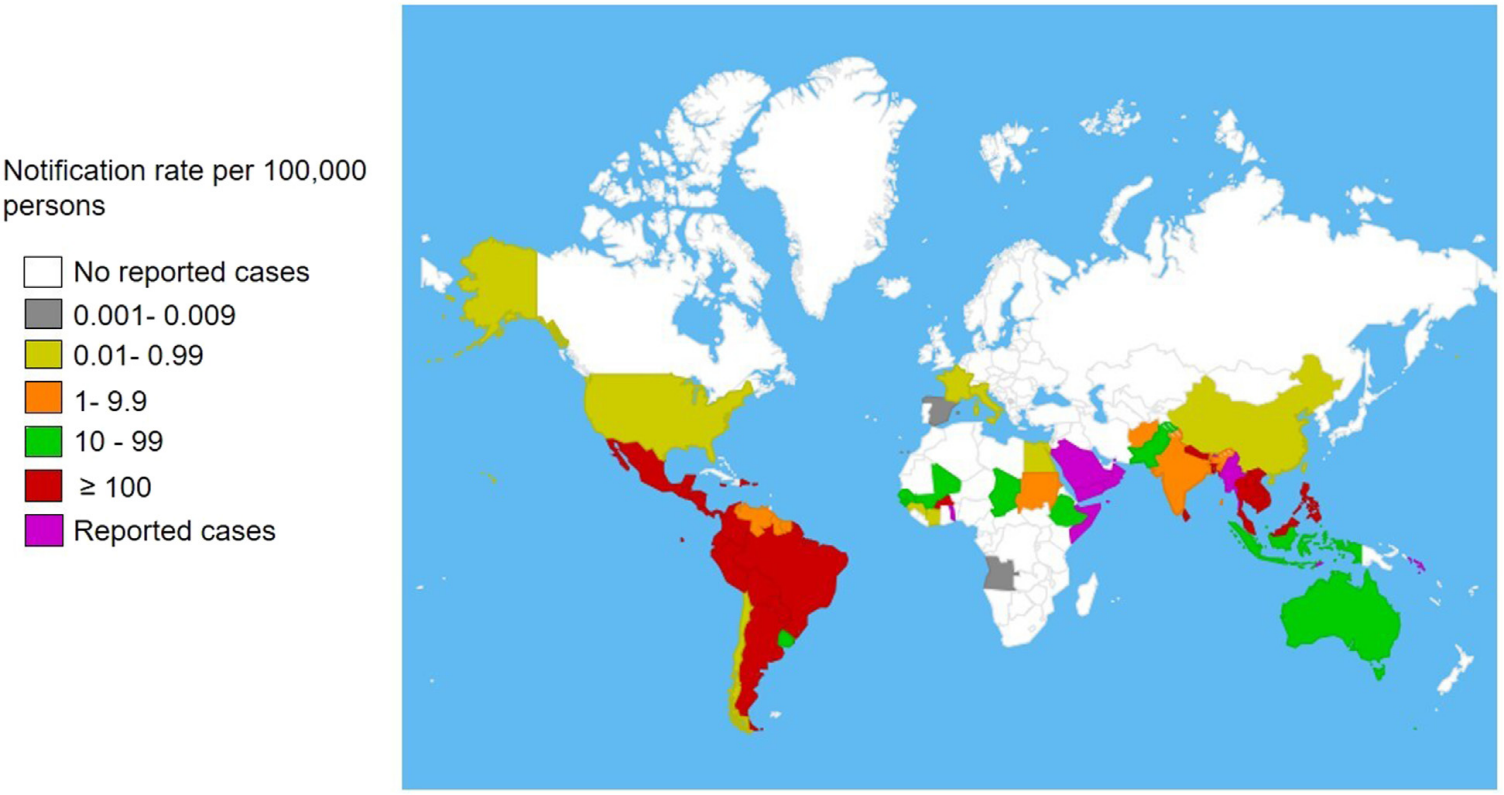
Countries reporting autochthonous dengue cases (November 2022-November 2023). Adapted from World Health Organization.^2^

The Americas reported nearly 80% of these cases, with 4.1 million new cases, surpassing the 2019 record by over 1 million. Additionally, 6710 were severe, resulting in 2049 deaths. However, the actual extent may be underestimated as most primary infections are asymptomatic, and dengue reporting is optional in many countries. The World Health Organization estimates that about half of the world’s population is at risk of dengue.^2^

Because of the 4 circulating dengue serotypes, individuals can contract the infection multiple times. Infection with a different serotype implies a significant risk of severe dengue. This scenario is alarming in the Americas because of the simultaneous circulation of all serotypes, the lack of specific treatments, and the fact that 11% of Latin American cases require hospitalization.^1^^,^^2^

Historically, prevention has focused on vector control and avoiding mosquito bites. Fortunately, vaccine development has expanded strategies to prevent symptoms, severe cases, and hospitalizations.

The Centers for Disease Control and Prevention recommends Dengvaxia (CYD-TDV) for residents of endemic areas aged 9 to 16 years with confirmed previous dengue infection. The vaccine was protective against hospitalization and severe dengue in 79% and 84%, respectively.^3^ The European Medicines Agency approved Qdenga (TAK-003) for individuals aged ≥4 years. It reduced hospitalization because of dengue by 90%.^4^ Both vaccines are live-attenuated and contraindicated for immunosuppressed patients. Qdenga was approved in Argentina a year ago.

Thousands of patients worldwide are undergoing treatment with biologic drugs for dermatologic diseases. The challenge is to agree on the vaccination implementation, especially for patients undergoing biologic treatment with a previous dengue infection.

Psoriasis management guidelines recommend discontinuing biologics before attenuated virus vaccination. Experts disagree on the duration of suspension, suggesting avoiding immunosuppressant between 2 or 3 drug half-lives before and after immunization, or suspending for 4 weeks and resuming 1 to 2 weeks after vaccination.^5^

While experiences with yellow fever or measles vaccinations involve a single dose, the question arises for 3-dose schedules such as Dengvaxia (0, 6, and 12 months) and Qdenga requiring 2 doses (0 and 3 months).^3^^,^^4^

Argentina is a large country, with diverse climates. In colder regions, suspending biologics during winter for vaccination could be feasible. However, this scenario differs from subtropical areas were viral circulation is observed throughout the year.

Without robust evidence, consensus is needed swiftly, considering risks and benefits for immunosuppressed patients exposed to dengue in endemic areas until inactive or subunit vaccines demonstrating efficacy and safety become available.

## Conflicts of interest

None disclosed.
